# “*We should be at the table together from the beginning”:* perspectives on partnership from stakeholders at four research institutions in sub-Saharan Africa

**DOI:** 10.1186/s12939-022-01707-3

**Published:** 2022-08-17

**Authors:** Shirine Voller, Chama-Chiliba Miriam Chitalu, Alinane Linda Nyondo-Mipando, Timothy Opobo, Clare Ahabwe Bangirana, Nicki Thorogood, Joanna Schellenberg, Primus Chi

**Affiliations:** 1 Epidemiology and Demography Department, KEMRI Wellcome Trust Research Programme, Kilifi, Kenya; 2 Institute of Economic and Social Research, University of Zambia, Lusaka, Zambia; 3 Department of Health Systems and Policy, School of Global and Public Health, Kamuzu University of Health Sciences (Formerly College of Medicine), Blantyre, Malawi; 4 The AfriChild Centre, College of Humanities and Social Sciences, Makerere University, Kampala, Uganda; 5 Faculty of Public Health and Policy, London School of Hygiene and Tropical Medicine, London, UK; 6 Faculty of Infectious and Tropical Diseases, London School of Hygiene and Tropical Medicine, London, UK; 7 Health Systems and Research Ethics Department, KEMRI Wellcome Trust Research Programme, Kilifi, Kenya

**Keywords:** Global health research partnerships, Equity, Partnership principles, Partnership guidelines

## Abstract

**Background:**

Global health research partnerships have been scrutinised for how they operate and criticised for perpetuating inequities. Guidance to inform fair partnership practice has proliferated and the movement to decolonise global health has added momentum for change. In light of this evolving context, we sought in this study to document contemporary experiences of partnership from the perspective of stakeholders in four sub-Saharan African research institutions.

**Methods:**

We conducted qualitative interviews with 20 stakeholders at research institutions in four countries in anglophone eastern and southern Africa. Interview questions were informed by published guidance on equitable research partnerships. Data was analysed through an iterative process of inductive and deductive coding, supported by NVivo software.

**Results:**

Early-career, mid-career and senior researchers and research administrators from four sub-Saharan African research institutions described wide-ranging experiences of partnership with high-income country collaborators. Existing guidelines for partnership provided good coverage of issues that participants described as being the key determinants of a healthy partnership, including mutual respect, role clarity and early involvement of all partners. However, there was almost no mention of guidelines being used to inform partnership practice. Participants considered the key benefits of partnership to be capacity strengthening and access to research funding. Meanwhile, participants continued to experience a range of well-documented inequities, including exclusion from agenda setting, study design, data analysis and authorship; and relationships that were exploitative and dominated by high-income country partners’ interests. Participants also reported emerging issues where their institution had been the prime recipient of funds. These included high-income country partners being unwilling to accept a subordinate role and failing to comply with reporting requirements.

**Conclusions:**

Insights from stakeholders in four sub-Saharan African research institutions suggest that contemporary global health research partnerships generate considerable benefits but continue to exhibit longstanding inequities and reveal emerging tensions. Our findings suggest that long-term support targeted towards institutions and national research systems remains essential to fulfil the potential of research led from sub-Saharan Africa. High-income country stakeholders need to find new roles in partnerships and stakeholders from sub-Saharan Africa must continue to tackle challenges presented by the resource-constrained contexts in which they commonly operate.

**Supplementary Information:**

The online version contains supplementary material available at 10.1186/s12939-022-01707-3.

## Background

Both the benefits of and imbalances within global health research partnerships have been extensively documented. Imbalances include differential access to funding, knowledge, networks and educational opportunities [1, 2] and high-income country (HIC) research institutions have historically exerted greater power and influence than their low- and middle-income country (LMIC) counterparts. This has manifested in a variety of ways: HIC partners have set the research agenda [3–6], pursued interests which may not reflect LMIC partner priorities [7–9], dominated all stages of the research lifecycle from design [10] through governance and administration [11–15] to publication [1, 11, 16–19], and confined LMIC partners to operational roles [1, 17].

Guidelines for good partnering offer direction towards addressing inequities and guidance has proliferated in the global health and development sectors over the past 30 years. Table 1 lists a selection of these guidelines.

**Table 1:** Examples of partnership guidelines and resources

Bridging research integrity and global health epidemiology (BRIDGE) guidelines [20, 21]
TRUST global code of conduct for research in resource poor settings [22]
KFPE guide for transboundary research partnerships [23]
Canadian Coalition for Global Health Research partnership assessment tool [24]
Research Fairness Initiative implementation guide [25]
Rethinking Research Collaborative promoting fair and equitable research partnerships to respond to global challenges [26]
Rethinking Research Partnerships discussion guide and toolkit [27]

A recent scoping review of guidelines for ‘North–South’ research partnerships [28] identified 22 sources of guidance. The most prevalent topics were: partner roles, responsibilities and ways of working—which encompassed communication, transparency, and mechanisms for conflict resolution and decision-making, capacity strengthening, motivation and goals, resource contributions, agenda setting and study design, governance structures and institutional agreements, dissemination, national relevance, data handling and ownership, and funding. Other efforts to synthesise partnership guidance [29, 30] indicate strong concordance on the topics that stakeholders are encouraged to address, though there is limited evidence about the extent to which guidelines are used in practice. Guidelines typically focus on things that individuals and institutions should change. However, they may not always fully acknowledge the structural barriers and competing interests that get in the way of these changes being realised. Of note, *Nature Portfolio* recently committed to improving inclusion and ethics in its journals [31], informed by the *Global Code of Conduct for Research in Resource-Poor Settings* [22]. This is an encouraging illustration of how guidelines are being put into action and it complements similar initiatives by PLOS and others calling for greater equity in academic publishing [31]. There is growing demand for change and particular emphasis on ‘decolonising’ global health [32–34], which has been defined as ‘a movement that fights against ingrained systems of dominance and power in the work to improve the health of populations, whether this occurs between countries, including between previously colonising and plundered nations, [or] within countries’ [35]. Sceptics argue that until fundamental change is realised, however, including updating systems of reward and recognition, channelling more funding directly to LMIC country stakeholders [15] and going so far as an entire ‘systemic overhaul’ [32] (p1) that involves ‘dismantling of structures that preserve power’ (p1), partnerships will remain inequitable.

This study explored the relationship between principles of equity and practice in global health research partnerships by documenting the experiences of stakeholders at research institutions in sub-Saharan Africa. It used a broad definition, informed by Bradley [36], whereby global health research partnerships encompassed ‘the wide variety of arrangements that link researchers and research institutions in the global North and South’ (p3). The study enquired into what sub-Saharan African stakeholders considered to be the benefits of working in partnership with HIC research institutions, what made partnerships work well, what was problematic and the extent to which they felt partnerships were fair. The study was intended to provide a contemporary view on partnership from a range of sub-Saharan African stakeholders’ perspectives and to consider whether there was any evidence of changes which might reflect shifts in the dynamics of the system of global health.

## Methods

This was a qualitative study informed by a scoping review of the literature on principles and guidelines for ‘North–South’ research partnerships [28]. Semi-structured interviews were conducted with key informants from a sample of research institutions in anglophone eastern and southern Africa. Since the researcher conducting primary data collection was affiliated with London School of Hygiene and Tropical Medicine (LSHTM) and had access to a list of LSHTM’s international partners, a pragmatic choice was made to identify a sample from the list of institutions that had an active collaboration with LSHTM. The sampling approach was purposive with the intention of achieving diversity across the institutions sampled in terms of geographic location, type of institution (e.g. University, non-governmental organisation (NGO), independent research institute, national public health research institute), maturity as a research institution (for which the duration of the collaboration with LSHTM was used as a proxy), and scale of research activities (for which the size of the grant portfolio with LSHTM was used as a proxy). Selecting institutions in different geographical locations was intended to reduce the potential cultural bias of any single country. Seeking diversity in type of institution, institutional maturity and scale of research activities was an attempt to incorporate differences in domains that may affect partnership equity. For example, universities typically have considerable bureaucracy which affects organisational agility when working in partnership, while smaller and newer organisations may have more limited capacity which can contribute to power differentials in partnerships. Discipline and type of research were not included as selection criteria since an assumption was made that institutions would conduct research across a range of disciplines and types.

Institutions were contacted sequentially between April and August 2021 and data was collected between June and December 2021. The final sample comprised four institutions in four countries: Malawi, Tanzania, Uganda and Zambia. One was a small NGO with a strong research interest, one an independent research institute and two were Universities. Two of the institutions had collaborated with LSHTM for over 20 years while two had become partners within the last five years. The size of the active grant portfolio with LSHTM varied from under £100,000 to over £1 million across the four institutions. While all institutions were active partners of LSHTM, the researcher who collected data for the study had no prior relationship with any of them.

Key informants were identified by asking the key contact at each institution for a list of colleagues who had experience of working with HIC partner institutions. We requested that the list included staff in academic and research administration roles at varying levels of seniority but did not place any conditions around age distribution, gender or ethnicity. In two institutions, we also used snowballing to a limited extent to identify additional participants.

Two interview topic guides were developed: The first was informed by a scoping review of principles and guidelines for research partnerships [28]. The second topic guide was less detailed and contained broad questions to prompt participants to describe their experiences of partnership. Initially, the intention was to use the detailed topic guide with participants closely involved in individual partnerships and the high-level topic guide for interviewees less involved in individual partnerships who had a managerial or central administration role. However, through piloting and early interviews it was found that the high-level topic guide was often sufficient to elicit rich responses about a range of partnership issues. Questions from the detailed guide were used where additional prompts were needed. The topic guides were used flexibly given participants’ varying experience of different aspects of partnership. Interviews focused on the areas that each participant had most to comment on. Participants were invited to reflect on their experiences of partnership with any HIC partner, not only LSHTM. The interview guides used for data collection are included in Additional file 1.

Interviews lasted between 45 and 60 min and all were conducted in English using the web-based Zoom platform (Zoom.us licenced education version). Field notes were written after each interview to complement the transcript. The recording and auto-transcription functions of Zoom were used to record and generate a preliminary transcript from each interview. The final version of the transcript was produced by listening back to the audio-recording and correcting errors in the auto-transcript. Transcripts were anonymised at this point so that only the audio-recording and a password-protected participant masterfile contained participants’ names. The participant masterfile also included a unique reference for each interviewee and their contact details. Quotes used in the results incorporate the reference and role for each interviewee, e.g. *A02_EMR* indicates that the quote was from a participant from institution A who was an early or mid-career researcher (EMR). *C03_SA* indicates a quote from a participant from institution C who was a senior administrator (SA).

NVivo release 1.6 1121 was used to code interview transcripts and support data analysis using a combination of inductive and a priori coding in an iterative and exploratory manner. Initially, a sample of transcripts was coded inductively, first by hand and then with codes set up in NVivo. The data were then reviewed again and organised using a small number of broad categories. Transcripts were also coded deductively using the framework of the key themes arising from a scoping review of principles and guidelines for partnership [28].

## Results

Interviews were conducted with four to six participants within each of the four institutions included in the sample. In total, 20 interviews were conducted between 16 June and 7 December 2021. Interviewee characteristics are summarised in Table 2. Career stage was assigned as *Senior* where a participant held a position at Assistant Director or Director Level or Professor on the academic career path or had more than 15 years of experience within the organisation. Career stage was assigned as *Early or mid-career* for all other participants, i.e. those whose job level and experience did not meet the threshold for Senior. One third of participants met the criteria for Senior career stage, while two thirds met the criteria for early or mid-career. Six participants were female while 14 were male. Gender was not used as a selection criterion, and the unequal distribution of participants may reflect bias in the research sector at large towards employment of more men than women, though we were not able to ascertain this from the study.

**Table 2:** Participant characteristics

Gender	
Female	6
Male	14
Role type	
Management/administration	5
Research	15
Career stage	
Early or mid-career	12
Senior	8
Institutional affiliation	
Research institute	6
NGO	5
University	9

Interview findings are presented below under broad categories describing the benefits of partnerships, features that made partnerships work well, problems experienced in partnerships and fairness in partnerships with HIC collaborators. Since naming HIC collaborators may risk compromising participants’ confidentiality, we have not identified HIC institutions by name. It is worth noting, however, that participants drew on experiences of partnership with institutions in Canada, Norway, Spain, Sweden, Switzerland, UK and USA. In most of these countries, more than one research institution was named in the examples given by participants.

### Benefits of partnership with HIC research institutions

#### Capacity strengthening

The most widely reported benefit of engaging in partnerships with HIC research institutions was capacity strengthening. Benefits to individuals included PhD training, career development and improved skills and knowledge in scientific disciplines, research methods, grant management and administration. These benefits were gained through supervision, formal training, mentorship and on-the-job learning by interacting with collaborators, e.g.:

“When you are being engaged you can observe…the creation process, whether it is a creation of knowledge…of a grant, you participate and you see, so you build your skills on how to navigate around different calls. If there is any funding call, at least you know how to start.” [C05_SR].

PhD training was also seen to have strengthened institutional capacity through creating a *“pool of scientists”* [C04_SR], many of whom were reported to have progressed to senior leadership and management positions in participants’ own or other African institutions. Other examples of institutional capacity strengthening included support to establish a PhD programme and investment in research infrastructure, such as a laboratory.

#### Funding for research

Access to funding was the second most frequently cited benefit of working in partnership with HIC research institutions. Participants gave examples of how partnering with HIC collaborators had enabled them to access funding which they would not otherwise have been eligible to apply for because of funder restrictions, and had given them a higher chance of success because of the reputation of the HIC collaborator, e.g.:

“If you are trying to win a large grant, I am sure you have to demonstrate that you have the capacity to do the research. So if we were to bid for such grants as the prime [applicant] or on our own, where there is a requirement for lab capacity or other forms of capacity, then I’m sure we would not have had the research portfolio that we have now.” [B02_EMA].

#### Other benefits

Other benefits that participants reported included: exposure to opportunities, entry into networks, and visibility to funders which might lead to future grant funding, e.g.:

“You want to partner with others because it also helps you to be within the community of the same people who are working over the same things and it increases your influence and net worth.” [B01_EMR].

A couple of participants described how partnerships enabled researchers to fulfill the career goals and promotion criteria within their own organisations, such as grant income and publication. While most participants described how they and their institution had benefited from partnerships with HIC collaborators, several also talked about benefits to their country, including an enhanced international reputation for research leading to future funding, better health service provision and greater use of evidence-based decision-making where policy makers had seen the value of using research data to inform their policy choices, e.g.:

“Now I think there is an interest from policymakers in terms of ‘what evidence are you providing after doing an intervention or a study? What works?’” [D03_EMR].

### What made partnerships work well

#### Mutual respect

A number of participants described how mutual respect and appreciation of one another’s contributions were fundamental to the functioning of a partnership. Participants had a range of expectations about the extent to which partner inputs should be equal. Some advocated for full equivalence while others were satisfied with a smaller input where the HIC institution was the lead partner, provided that their own contribution was recognised, e.g.:

“Coming into the partnership with the attitude that…everybody has something to offer. It may not be equal. but just having that attitude that…everybody going into it has something to bring onto the table. I think is a very critical aspect in determining how the partnership is going to flow.” [A05_SA].

#### Early and continuous involvement

Many participants commented on the importance of having an input at all stages of a project from conception through to design, implementation, analysis and writing up. Particular emphasis was placed on being involved early on in order to be able to influence design and budget allocation, e.g.:

“If we are really partners then we should be sitting at the table together from the beginning, all the way through the budgeting, so that it's fair across the line.” [C02_EMA].

#### Role clarity

A number of participants felt that reaching clarity on the roles and responsibilities of the institutions and individuals within a partnership was important for the partnership to function well. Participants felt that responsibilities should be established through joint discussion and boundaries respected once roles has been agreed e.g.:

“I'm always very, very keen on ‘let's be clear on what the roles are and what is expected and what we each are supposed to achieve’, so that there is no misunderstanding and nobody ends up feeling short-changed.” [D01_EMR].

Some participants had a preference for formal documentation such as terms of reference, Memoranda of Understanding or documented principles for collaboration and conflict resolution. Others emphasized the benefits of an informal agreement on the principles for working together, including retaining flexibility for roles to evolve as the partnership developed.

#### Experienced collaborators

Several participants described how it was easier to work with HIC partners who were experienced in working in low-resource settings, understood the constraints of the context and were willing to adapt their own systems and requirements to fit the needs of their partner, e.g.:

“When you're working with … experienced collaborators they’ve got mechanisms to start asking about things…way ahead of time… so they do anticipate that things can go wrong, and they know how to communicate.” [B04_SR].

Participants felt that experienced partners were typically more flexible, more engaged in helping to solve problems and more sympathetic to external constraints than inexperienced HIC collaborators.

#### Effective communication

Several participants commented on the importance of communication between partners that was frequent, timely, transparent and two-way. Where communication worked well it was felt to lead to a shared vision about the purpose of partnership and each partner understood what the other wanted to get out of the relationship. The ability to discuss issues and address them openly and respectfully, for example, in relation to budget allocation, was seen to be critical, e.g.:

“If there are issues that, you know, perhaps we need to deal with, or that we were not comfortable with, we must be able to sit as partners and talk about them, rather than one of the partners being the partner, at the same time, the Court.” [A03_EMR].

#### Long term relationships

A couple of participants talked about the importance of a long-term relationship that transcended individual projects, generated institutional benefits and left a legacy for the future, e.g.:

“We should also remember that we need to strengthen this department as part of the capacity building within this project, so that level of consideration is also, it’s beyond the research. To make sure you will also leave a footprint after the research is done.” [B01_EMR].

Long-term collaborations allowed for trust and understanding between partners to develop which improved the working relationship and for initiatives such as faculty exchange and joint post-graduate training programmes to be established.

Several participants talked about specific ongoing or past collaborations which exemplified many of the themes of good partnership practice.

Example of good partnership practice A HIC institution leading a grant application approached the sub-Saharan African collaborator at concept design stage to solicit input on study design and agree outline budget requirements. The application was a success and at each subsequent stage of project set-up, implementation, analysis and writing up the sub-Saharan African collaborator was fully involved. The intended project beneficiaries were also involved as peer researchers and were consulted on key decisions. Roles and responsibilities and a communication structure for the project partners were agreed early on. The HIC partner offered suggestions and provided support in areas in which the sub-Saharan African lacked experience and the sub-Saharan African partner gave direction on issues where they had more expertise. The sub-Saharan African partner had the autonomy to use their budget flexibly to meet the project needs as it evolved and formal reporting was minimised while informal communications were frequent and two-way. Overall, the sub-Saharan African partner felt that they had as equal a stake in the project as the HIC partner and were respected as equals. A relationship of trust and respect developed and the project led to other collaborative initiatives between the institutions.

### Problems of partnering with HIC collaborators

#### Late involvement and confined role

A number of participants described the frustration of being asked to join a partnership after key decisions about project design and budget allocation had already been made. This frustration was exacerbated when their roles had remained limited throughout the collaboration, they had little influence on decisions and their involvement was diminished at key stages of the research process, particularly during data analysis and publication.

“They wanted to treat us as research assistants and not as partners in a developing country context…When it came to authorship, they wanted to be the ones who determine who was to participate.” [D02_SR].

Participants described a range of experiences with respect to data ownership and access to data. Some felt that shared ownership and rights to use data by the institution that generated it were typically clear and they had had no concerns, while others had experienced difficulties in accessing and using data even when they had been involved in generating it. Several participants described having been excluded from the writing process or the HIC partner demanding senior authorship of papers even when their contribution did not justify it, e.g.:

“For me, it’s very demeaning when you are passed over for an opportunity to co-author on work you conceptualised from scratch and you were available, because a student somewhere has only come in to analyse the data.” [A01_SR].

#### Exploitative relationships

Several participants had experienced partnerships where they felt that benefits were unevenly distributed between partners in favour of the HIC partner, the relationship was exploitative and HIC stakeholders had prioritised their own objectives over those of their partners, e.g.:

“You’re more on the receiving end and you sometimes question and feel, ‘Am I only being used?’ To just meet the interest of somebody else.” [A05_SA]

Participants proposed that there needed to be greater recognition of all contributions and that HIC institutions should offer benefits to their partners to balance out the benefits they had accrued from the relationship.

#### HIC partner superiority

Several participants described experiences of HIC partners behaving in a supercilious manner, lacking humility and not acknowledging their partners’ competence. Participants described how HIC partners often attributed greatest value to the contributions they brought themselves, such as funding and the research capacity of their institution. Two participants commented on how HIC partners failed to acknowledge that their institutions’ reputation and success was to a large extent based on work that was only possible because of working in partnership with LMIC partners, e.g.:

“I think that our northern partners or Western European partners have been a little bit slow to realize two things: one is the historical predisposition that has created… a lopsided system, where one person is seen to be more important or cleverer. A lot of these sort of historical predispositions have nothing to do with innate ability. They've also failed to realize that a lot of their own growth is the result of these partnerships and that there is probably more they are gaining from the partnerships than the so-called Southern partners are gaining.” [C01_SR].

#### Inauthenticity

Several participants had experienced a disconnect between a HIC partner’s rhetoric of equality but practice of inequality, for example if a project was not going in the direction the HIC partner expected. In several examples, the HIC institution had used their position as the lead partner to *“bulldoze”* [B04_SR; D03_EMR] their way forward, even when this contravened a prior agreement about roles and responsibilities. For example:

“Our bargaining power is always to a certain extent [limited]…you reach a certain point, whereby if they say, “This is how things should be done”, you bow down to that.” [B03_SR].

One participant gave an example of a HIC partner using capacity strengthening as a selling point in a grant application, yet when the project was implemented, no capacity strengthening was offered. Another described feeling misled by a HIC institution that had framed a project as a collaborative venture yet issued a consultancy contract which positioned the sub-Saharan African institution as a service provider. This had disadvantageous tax implications and left them with little room for intellectual contribution or rights to use the data:

“The attitude is that you don't know it, and they know it all, and so your responsibility…is to follow direction and not to contribute alternative views and where you contribute alternative views they are shot down.” [A05_SA].

#### Micro-management

Several participants gave examples of where HIC partners had micro-managed research projects, overstepped the boundaries of their role as lead partner and interfered in the sub-Saharan African institution’s operations. For example, one participant described how they had been required to send documents for the HIC partner to review and were expected to attend meetings which were framed as progress meetings, but whose purpose seemed to be for the HIC partner to monitor their activity and control operational decisions. Several participants alluded to HIC partners having an attitude of entitlement, encapsulated in this comment:

“…they go in, like IN. It’s like when you enter the house and you are invited to sit in the sitting room, someone can go up to the bedroom.” [C03_EMR]

#### HIC partner failure to accept a subordinate role

Several participants described challenges where their institution had been the lead partner and had sub-contracted to a HIC institution as part of a collaborative project. They had found that some HIC institutions had been resistant to accepting a role other than as the lead partner and failed to submit financial and technical reports to the standard requested, e.g.:

“The resistance was there initially in terms of them [HIC partners] being at the mercy of the Southern partner in terms of the Southern partner determining…what support they needed … and the amount of funding that could be made available for that support.” [D01_EMR].

Participants felt this was wrong given that the reporting requirements were very similar to those that their institutions were expected to comply with when sub-contracted by a HIC institution.

#### Other problems

Other problems that participants had experienced included: slow contracting, delays in payment and inflexibility and lack of support from HIC partners, especially where the HIC partner did not understand the challenges of the context in which they were operating, e.g.:

“Our partners in the higher income institutions may not actually understand that what appears to be a very simple task to them may not necessarily be a very simple task for us.” [B02_SA].

One participant commented that most successful health research institutions in sub-Saharan Africa had a long-standing relationship with a HIC university or research institution and senior staff often had a joint appointment. While ostensibly beneficial, he felt that this also presented challenges: staff in leadership positions may be compromised by seeking to meet the expectations of the HIC institution, which might be in conflict with the interests of the African institution and limit its trajectory towards independent success.

### Fairness in partnerships with HIC collaborators

The concept of fairness underpinned many of the issues that participants raised about partnership with HIC collaborators and was also discussed explicitly. A couple of participants commented that inequities existed in partnerships between organisations in the region, not only in relationships with HIC collaborators, and attention should also be paid to these. However, this theme was not explored in detail in this study. Participants typically described having experienced both fair and unfair partnerships with HIC collaborators and partnerships that had elements of fairness and unfairness. Most striking was participants’ initial responses to the question of whether partnerships were fair. Many laughed at the question and paused before giving an answer. Some participants implied that the complexity of the concept made it a difficult question to address succinctly, several others suggested that it was futile to isolate the issue and make a judgement on fairness in partnerships given the pervasiveness of unfairness across many aspects of life, while others implied that the pursuit of fairness was a luxury that was beyond practical consideration, e.g.:

“I think fairness becomes an abstract thing here. You do what you have to do to keep running sometimes.” [A01_SR]

#### Funding

The topic of funding generated most discussion with respect to fairness and elicited a range of views. One participant felt that it was reasonable that when funding originated from a HIC, the institution from that country should be the lead partner, while a contrasting view was that funding calls should always be open to applicants from any country. Another participant felt it was fair that when funding was derived from HIC taxpayers a substantial share of this funding was retained and invested in the HIC where the funds originated. Another participant described a recent situation where their institution had been in competition with others in the region to find a partner for a call requiring a UK lead applicant. They felt that this requirement was unfair because despite having a strong proposition they had not been able to apply as there were no UK partners left to partner with.

Several participants were critical of funders with low overhead limits which meant that their institution had to subsidize projects. Two participants described their experience of the distribution of funds between LMICs and HICs being unfair: one grievance was that majority share of the funding often remained in the HIC institution when the work largely took place in LMICs. The other grievance concerned salaries and benefits going to HIC institutions that were disproportionately high in comparison with the compensation that their own institution’s staff received, even after having accounted for cost-of-living differences.

Several participants described how feelings of mistrust and inequity were fueled when the lead partner lacked transparency about how funds had been allocated between institutions. In contrast, several participants felt that as long as their institution received sufficient funding to deliver their component of the work, this was fair, and they were not concerned about how much money the lead partner received, e.g.:

“I really don't care how much money is going to the UK, because I know that I've got enough funds to do this study.” [B04_SR]

One participant commented on how responsibility lay with his own institution to pay close attention during budget development and to negotiate a fair funding allocation at the outset and that problems only arose when insufficient care was taken at this stage. A couple of participants had experienced receiving inadequate funds to deliver the work expected of them, and—having agreed to a scope of work – had been expected to take on additional work without any extra funding, e.g.:

“When you look at it and the expectation, it is somebody asking you to deliver a Rolls Royce and they're giving you money to buy a Toyota.” [A05_SA]

Two participants felt that the high Masters’ tuition fees charged by HIC universities to LMIC students were unfair when these universities’ core funding and reputation were founded on work done in LMICs. One participant described how HIC partners had a duty to distribute the benefits when they had gained from work conducted in LMICs, and it was unfair when this did not happen. Another participant felt that the tone of a partnership was to a large extent set by funders. This individual felt that partnerships were more likely to be fair when the funder issued criteria for equitable participation than where arrangements were left to the lead partner to determine.

#### Capacity strengthening

Capacity strengthening was the second most frequently discussed topic in relation to fairness in partnerships. Several participants described their expectation that capacity strengthening should be inherent in the design of research partnerships with HICs and when it was, this was fair. A couple of participants felt it was unfair when their expectations with respect to capacity strengthening were not met. For example, one participant described how their institution had strengthened the capacity of a HIC partner when they felt it should have been the other way around. One participant felt that when capacity strengthening was narrowly focused, e.g. on PhD training, individuals remained dependent on HIC partners because they were not exposed to the broader experiences, skills and capabilities needed to become a successful independent researcher. These included grant writing skills, how to engage with funders and networking skills, e.g.:

“You really don’t know how that process of engagement goes when you're always in the lobby when everyone else is in the conference room.” [C02_EMA]

Two participants felt that HIC partners intentionally restricted opportunities for capacity strengthening because this protected their own position in the partnership hierarchy, and they questioned the commitment of HIC partners toward supporting LMIC researchers’ independence. The inverse of this experience was described by a participant who had been encouraged by the principal investigator from a HIC partner to write grant applications, supervise students and participate in training. The participant described the relationship as being very fair.

One participant differentiated between individuals’ responsibility to negotiate a fair relationship with one another and structural unfairness which was difficult to tackle as an individual. Several other participants alluded to a blurring of the boundaries between individual and systemic inequity. For example, one participant described how individuals from HIC institutions were inclined to perpetuate systems (systemic inequity) which supported their own career advancement (individual inequity), e.g.:

“They work within this system that is designed in a super-biased way and somehow these well-meaning people are unable to come out of this. In some cases they might even be tempted to use this system to survive. To get a favour.” [C01_SR].

Several participants described the unfairness of being limited by the HIC partner in the extent to which they were able to contribute to decisions relating to project design and delivery, while two others described feeling exploited by a HIC partner who had restricted their involvement in the partnership to data collection and excluded them from analysis and publication, e.g.:

“I have worked on studies where I knew I could contribute more, but your role is already defined: ‘You are managing fieldwork, you are recruiting and overseeing data collectors, and sending us the data’. End of story. I’m like, ‘I want to be involved in the analysis, it’s qualitative data, I am excited about these things, I want to be involved and maybe co-author’, but that option is not provided many times.” [A01_SR].

One example was given where a participant’s institution had been running a joint PhD programme with a HIC university. When the Memorandum of Understanding for the arrangement expired the HIC partner had decided unilaterally that the programme should not be renewed but should become a dual PhD programme whereby students could register at either institution. The HIC institution promoted this new arrangement as a benefit, but the participant felt that it was disadvantageous because the best students who could secure sponsorship chose to register at the HIC partner institution because of its strong reputation. Their own institution missed out on being associated with the highest calibre candidates.

## Discussion

Interviews with early to mid-career and senior researchers and research managers at four institutions in anglophone eastern and southern Africa revealed wide-ranging experiences of partnership with HIC collaborators, both positive and negative. Existing principles for partnership [28–30] provide good coverage of the issues that participants described as being the key determinants of a healthy partnership, for example: mutual respect, involvement of all partners from concept design stage throughout the research lifecycle, clear governance and open communication. A small number of participants referred to published partnership guidelines, but none mentioned having used them. There is scope for institutions to adopt and adapt existing guidelines, and it would be useful to probe further into why, after several decades of guidelines being available, they are rarely used.

Participants described a range of benefits of partnering with HIC institutions. Foremost among these was capacity strengthening. Mutual learning and capacity exchange have been promoted in partnerships to acknowledge the value that each partner brings to the table [23, 37] and it would be interesting to explore the extent to which HIC partners also identify capacity strengthening as a benefit. Another key benefit identified was access to research funds, partly as a consequence of restrictions on LMIC organisations applying directly for funds originating in HICs. As discussed below, there is some evidence that more funding is being granted directly to LMIC institutions. This might alter what benefits LMICs perceive to be gained from partnering with HICs in future.

Meanwhile, participants had recently experienced a wide range of partnership inequities that have been well documented in the literature. This finding suggests that there is still some way to go before principles of fairness are embedded in practice and is consistent with claims that the system of global health remains colonial at its core [33]. Inequities experienced by participants included only being invited to participate in a study after the research concept and design had been determined [10], being offered only a limited role [1, 17], receiving fewer benefits than HIC partners [36, 38], HIC partners interfering in the LMIC institution’s operations [12, 39] and HIC partners over-claiming authorship positions [16, 18]. Several less well documented challenges also emerged which had arisen when LMIC institutions were in the lead partner role. For example, one HIC institution was unwilling to adhere to the sub-contracting requirements issued by a lead partner from sub-Saharan Africa. Another was dissatisfied when the funder re-routed how funds were channeled so that they flowed from the funder to LMIC partners who then commissioned the support they needed from HIC partners. The experiences of sub-Saharan African partners when leading partnerships have had little coverage in the literature to date. They are likely to become more prevalent as funding patterns change and partnership structures evolve towards more partnerships being led from sub-Saharan Africa.

The narratives of participants from institutions with more limited capacity for research and research management hinted at some differences in the issues they were experiencing in comparison with participants at institutions with greater capacity and depth of resources. This is not something we have seen explored in detail elsewhere. For example, participants who felt that they and their institution were held back by lacking skills, experience or resources expressed a strong demand for capacity strengthening in science and operational areas. This appeared to be a lower priority for participants from high-capacity institutions for whom other issues were in the foreground, such as how to deal with HIC institutions who were unwilling to accept a subordinate position in a partnership.

Perhaps this differential underpins the finding that participants working in research institutions with greater capacity appeared to be able to exercise greater power and influence in their dealings with HIC partners than participants from institutions with more limited capacity. That is not to suggest that all power imbalances are a consequence of capacity differentials. However, many of the negative experiences that participants reported appeared to be related to their institution having less capacity than a HIC partner and this contributed to the power differential between them, as has been discussed elsewhere [40]. This reinforces the need to ensure that individuals and institutions in capacity-limited contexts continue to be supported to develop the skills and experience to compete in a global research arena, and that this is done respectfully. Historically, HIC technical partners have provided much of the support, leveraging funding from HIC governments, commercial and non-profit entities, and are likely to continue to play a significant role for some time to come. However, pressure is growing from advocates in LMICs [34, 41, 42] for LMIC governments to increase investment in research and move away from the reliance on foreign investment in research coined as neo-dependency [43]. Furthermore, emerging entities such as the African Academy of Sciences’ Alliance for Accelerating Excellence in Africa and Africa Centers for Disease Control and Prevention provide hubs of technical expertise, mechanisms to support capacity strengthening and channels through which funds, from any source, can be managed and distributed to address issues of regional priority.

There has been a steady crescendo of voices calling out the ills of the colonial legacy in global health and challenging the systems that perpetuate structural inequities and maintain the status quo where HICs dominate the discipline [35, 44, 45]. Though it has taken several decades to build momentum, we may be approaching a tipping point for a major re-evaluation of how global health is conducted: some funders have already diversified their approach or are re-considering how to invest, for example increasing direct funding to institutions in LMICs, of which several examples were given in this study. Groups such as the UK Collaborative on Development Research [46] and Council on Health Research for Development [47] provide platforms to share resources [48, 49], convene discussions and secure commitments to changing funders’ practice. Funders also have an influential role in setting expectations for how partnerships should operate and in choosing what to fund. Ring-fencing funds for activities that promote partnership development and increasing investment in institutional and systems strengthening are two possible options. In parallel with changes in the funding environment, powerful stakeholders in the ‘global South’ are increasingly acting as advocates for change [34] and HIC research institutions are starting to look critically at how they engage with partners. For example, the authors are aware of ongoing exercises at two UK universities specializing in global health to review their policies and practice in service of achieving more equitable partnerships, while a number of institutions have committed to undertaking self-assessments using the Research Fairness Initiative reporting tool [50]. Future research that captures the perspectives of HIC stakeholders on partnerships, including motivations to change practice and the challenges thereof, would make a useful contribution to the evidence base.

The findings from this study suggest that the downstream impacts of changes in ideology and policy are, to a limited extent, reflected in the experience of stakeholders in sub-Saharan African research institutions, but there remain significant barriers to overcome. Embracing change poses challenges to those who are faced with relinquishing power [51], and several participants in this study gave examples of HIC partners who had been reluctant to cede control, speculating that this was driven by fear of losing the opportunities on which their careers and reputations had been built.

Notwithstanding the negatives, almost all participants in this study commented on the considerable benefits that they had experienced themselves and the value added to their institutions and countries from working with HIC partners. The overarching sentiment was not a demand for HIC research institutions to exit the global health stage. Indeed, several participants commented that it would be an abdication of responsibility for stakeholders with access to resources and expertise not to use it to benefit others. Participants’ views on what still needs to change and how to achieve greater equity in global health partnerships represented a microcosm of wider discussions in the field and were largely optimistic that things are moving in the right direction.

## Limitations

Efforts were made to incorporate diversity in the characteristics of institutions included in the sample and to identify participants with a range of jobs and experience in order to provide a broad range of perspectives. However, the criteria for selecting institutions used proxy indicators for institutional maturity and scale of research activities which may not have been the most robust measures of these characteristics. We did not include a criterion to select for type of research or diversity across research disciplines, e.g. product development, clinical trials, basic science, social science and this may be a useful selection criterion for future studies. Including only institutions in countries where English is an official language, and excluding francophone and lusophone nations, is a further limitation of the study. In recognition of these limitations, we seek only to offer illustrative findings and do not claim that these are representative of the concerns of stakeholders at research institutions across anglophone eastern and southern Africa, let alone a broader geographical area.

## Conclusions

Evidence from stakeholders in a small sample of research institutions in anglophone eastern and southern Africa suggest that contemporary global health research partnerships generate benefits but continue to exhibit longstanding inequities and reveal emerging tensions. Published principles and guidelines for partnership seem to be relevant but are rarely used. Raising awareness of the existence of principles and guidelines alongside a commitment from stakeholders to adopt and adapt them may offer a useful step forward. The distribution of power between partners appears to be gradually levelling out as research institutions in sub-Saharan Africa grow in stature, research funding is re-configured and movements for research equity and decolonising global health gain momentum and drive change. Meanwhile, long-term financial and technical support targeted towards institutions and national research systems remains essential to fulfil the potential of research led from sub-Saharan Africa. As the landscape of global health changes, HIC stakeholders need to identify new roles in partnerships, and stakeholders from LMIC must continue to tackle challenges presented by the resource-constrained contexts in which they commonly operate.

## Supplementary Information

**Additional file 1.** Interview topic guides.

## Data Availability

The small sample size and nature of qualitative research makes it impossible for us to avail the data without compromising participants’ confidentiality or redacting transcripts to the extent that their meaning is distorted from that which informed the interpretation of the findings.

## Abbreviations

HIC: High-Income Country
LSHTM: London School of Hygiene and Tropical Medicine
LMIC: Low- and Middle-Income Country
NGO: Non-governmental organisation
