# Supplementary material for: “We should be at the table together from the beginning”: perspectives on partnership from stakeholders at four research institutions in sub-Saharan Africa
Source: Int J Equity Health. 2022 Aug 17;21:111. doi: 10.1186/s12939-022-01707-3 (PMC9387072; doi:10.1186/s12939-022-01707-3)
Supplement: Supplementary file 1 — Additional file 1. Interview topic guides. [file 12939_2022_1707_MOESM1_ESM.docx]

# **Additional file 1: Interview topic guides**

# Detailed topic guide

[Pre-amble]

**Participant’s role**

- Could we start by you briefly describing your current job? *[Prompt for details: How long in role, nature of role]*
- Have you had any other roles at X [name of research institution]? [*Prompt for details]*
- Did you work anywhere else before joining X? [*Prompt for details. Only ask question if started at X in last 5 years].*

**Experience of partnership**

Now I would like to hear about your experience of research partnerships with institutions in high income countries.

- Could you describe some of the recent partnerships that you have been involved in?

*[Who partners were, funder, ongoing or finished, purpose and scope, size $$, interviewee role in partnership, get sense of what interviewee understands partnership to be].*

- Now can you describe in more detail [project name]/ the partnership with LSHTM/a partnership with another institution that you have been closely involved with?

*[Probe for information about purpose and scope, institution(s) involved, funder, when started, respondent’s role and extent of involvement, role of his/her institution. Allow participant to talk at length].*

**Overall experience**

- Overall, what have been the most positive aspects of the partnership?

*[Probe: for self, for institution, for other stakeholders. Why and how?]*

- What has been difficult, or not worked well, and why?
- What would make the partnership work better?

*[Probe: if could change one thing about working with the high-income country partner, what would it be?]*

- ­ What are the most important factors in determining how well this partnership has worked?
- Do you think the partnership is fair?

*[Probe: for self? for institution? Why yes or why no, what fairness means, what is stopping it from being fair (if so), e.g. structural barriers such as (poor) education, is concept of fairness important?]*

- Is this partnership typical of other research partnerships that you have been involved in with high income country institutions?

*[Probe: how yes and how no, seeking examples and try to assess how widespread different issues are]*

**Themes of fair partnership**

*[Adapt or don’t ask questions if already covered in previous discussion]*

Now I will ask some more specific questions about the partnership you talked about previously, or a different partnership if you prefer.

*[If different, prompt for brief details]*

- How did you come to be involved in this partnership?

*[Probe: own and institution’s role in agenda setting, proposal writing, study design; how these phases went, whether topic a local – institutional & country- priority]*

- How were the goals of the partnership agreed on?

*[Probe: are own and institution’s motivations recognised and priorities reflected? Benefits to self and institution]*

- Is there any form of written agreement, like a memorandum of understanding or terms of reference, in addition to the contract?

*[Probe: how developed, whether useful, other governance structures? How decisions made?]*

- How are roles and responsibilities in the partnership agreed on? How do members of the partnership communicate and work with one another?

[*Probe: transparency, accountability, resolving conflicts, is there a steering group or advisory group?]*

- What does each partner contribute to the partnership?

*[Probe: value placed on different contributions, is there proper acknowledgement of own institution’s contributions and what it offers high income country partner? Are benefits proportionate to contributions?]*

- Can you say a bit about funding for the partnership?

*[Probe: how funds distributed and managed, whether funds adequate for the work – are full costs covered, plans for securing future funding, satisfied with funding arrangements? How much awareness of and involvement with funder?]*

- Has there been any capacity strengthening, or is any planned?

*[Probe: what and for whom? Individual, institutional? Research, research management? Formal, informal? Support for budgeting, contracting, ethics, research skills?]*

- Can you say a bit about how data is stored, shared and who uses and owns it?

*[Probe: are there agreements on data ownership and sharing, material transfer agreements etc. If so, how reached? issues experienced].*

- Is there a dissemination plan for the partnership? Are there plans to use the findings to inform policy and practice?

*[Probe: Types of outputs planned, audiences, authorship;*

*[Probe for policy & practice: How? By whom? Resources dedicated to this? Is this relevant?]*

- We have covered some of the key areas documented in guidelines for partnership. Are there other important aspects of this partnership that you would like to talk about?

*[Give space for participant to talk about other features of partnership]*

- Are any discussions happening about equitable partnerships in your institution, in networks that you are involved in?

*[Probe: where, who, what issues, how being taken forward]*

*­***Closing**

We are now approaching the end of the interview. Before we finish, is there anything else you want to say about your experience of partnership with high income country research institutions?

*[Prompt for details]*

Thank you very much for your time. I may come back for clarification if there is something that is not clear or if I have some additional questions as I am writing up the interview, if that’s OK? Please contact me at any time if you have any questions, comments or concerns.

*Close interview*

# High level topic guide

[Pre-amble]

**Participant’s role**

- Could we start by you briefly describing your current job? *[Prompt for details: How long in role, nature of role]*
- Have you had any other roles at X [name of research institution]? [*Prompt for details]*
- Did you work anywhere else before joining X? [*Prompt for details. Only ask question if started at X in last 5 years].*

**Experience of partnership**

Now I would like to hear about your experience of research partnerships with institutions in high income countries.

- Could you describe some of the recent partnerships with high income country research institutions that you have been involved in?

*[Probe: Role in relation to partnerships; Who partners were, funder, ongoing or finished, purpose and scope, size $$, get sense of what interviewee understands partnership to be].*

- Have you been involved in [project name]/ the partnership with LSHTM?

*[If yes, probe: how involved? How has experience been? What has been positive, negative, what could be better? What needs to change?].*

- What are the most positive aspects of working with high income country partners?

*[Probe: for self, for institution, for other stakeholders. Why and how?]*

- What aspects are difficult or do not work well?

*[Probe: for self, for institution, for other stakeholders. Why and how?]*

- What is needed to make partnerships with high income country partners work better?

*[Probe: if could change one thing about working with high income country partners, what would it be?]*

- Thinking about your experience of partnerships, what are the most important factors in determining how well a partnership works?
- ­Have the partnerships you have been involved with been fair?

*[Probe: for self? for institution? Why yes or why no, what fairness means, what stops partnerships from being fair (if so), is concept of fairness important?]*

- Are there discussions about equitable partnerships happening in your institution, in networks that you are involved in?

*[Probe: where, who, what issues, how being taken forward]*

*­***Closing**

We are now approaching the end of the interview. Before we finish, is there anything else you want to say about your experience of partnership with high income country research institutions?

*[Prompt for details]*

Thank you very much for your time. I may come back for clarification if there is something that is not clear or if I have some additional questions as I am writing up the interview, if that’s OK? Please contact me at any time if you have any questions, comments or concerns.

*Close interview*
